# A re-evaluation of the basicranial soft tissues and pneumaticity of the therizinosaurian *Nothronychus mckinleyi* (Theropoda; Maniraptora)

**DOI:** 10.1371/journal.pone.0198155

**Published:** 2018-07-31

**Authors:** David K. Smith, R. Kent Sanders, Douglas G. Wolfe

**Affiliations:** 1 Biology Department, Northland Pioneer College, Holbrook, Arizona, United States of America; 2 North Canyon Medical Center, Gooding, Idaho, United States of America; 3 White Mountain Dinosaur Exploration Center, Springerville, Arizona, United States of America; Università di Roma, ITALY

## Abstract

The soft-tissue reconstruction and associated osteology of the North American therizinosaurian *Nothronychus mckinleyi* is updated. The cranial nerve topology is revised, bringing it more in line with coelurosaurs. The trunk of the trigeminal nerve is very short, with an incompletely intracranial trigeminal ganglion, an ophthalmic branch diverging anteriorly first, with later divergences of the maxillomandibular branches, following typical pathways. The facial nerve has been re-evaluated, resulting in a very typical configuration with an extracranial geniculate ganglion. The single foramen leading to the cochlea probably transmitted the vestibulocochlear nerve, along with some fibers of the facial. This configuration is reduced from the more standard three foramina (vestibular, cochlear, and facial) and may be apomorphic for therizinosaurs. Some alteration is proposed for the dorsiflexive musculature. The insertion point for m. transversospinalis capitis is partially changed to extend onto the parietal, along with a proposed functional difference in the moment arm. The expansion of the basicranial pneumatic system is limited to the paratympanic system, enhancing low frequency sound sensitivity. There is little expansion of the median pharyngeal and subcondylar sinuses. Ossification of the surrounding epithelium may provide some information on the embryology of the theropod skull. It may be associated with a reduced stress field, or the general similarity of the basicranium with anterior cervical vertebrae may reflect activation of a cervical vertebral (Hox) gene regulating ossification of the pneumatic sinuses. This might be a local, selectively neutral, fixed gene in the basicranium reflecting embryological regulation of cervical vertebrae development.

## Introduction

Therizinosaurs were a lineage of unusual theropods from the Cretaceous of Asia and North America [1, 2]. They are sufficiently aberrant that their status as theropods was determined only thirty-five years ago [3]. Remains of these animals are usually quite rare in the fossil record, but they can be locally common [4, 5]. Various aspects of their paleobiology have been increasingly discussed as probably herbivorous theropods [6, 7, 8, 9, 10, 11, 12, 13].

*Nothronychus mckinleyi* was the first announced therizinosaur from the Upper Cretaceous of North America [14, 15, 16] from fluvial/flood plain deposits in the Turonian Moreno Hill Formation of western New Mexico. Subsequently a second species, *N*. *graffami*, was recovered from marine rocks of the Mancos Shale of southern Utah [17]. Additionally, starting in 2005, the basal therizinosaur *Falcarius utahensis*, from fluvial and overbank deposits of the Barremian Cedar Mountain Formation, Yellow Cat Member, Utah, was announced and described [4, 9, 12, 15].

This paper represents an update of the basicranial description of *Nothronychus mckinleyi* in light of new material and information. It presents some reinterpretation of the cranial nervous system and muscular systems (Table 1). The original pneumatic interpretation of Smith [10] is further supported using information based on the embryology of a chick [18], the associated incorporation of anterior cervical somites into the basicranium, and the resulting tripartite origin of the vertebrate skull. Therefore, the basicranium of therizinosaurs is structurally homologous with a cervical vertebra and the middle ear is derived from three separate components, as in modern birds [18]. Development of the skull and exaggerated pneumaticity patterns may have been related to genetic control of the anterior cervical somites and not stress fields. A qualitative interpretation of the influence of the pneumatic system on the sensory systems is presented with the result that the increased tympanic systems would result in very low frequency optimal sound reception, possibly extending to infrasound.

**Table 1:** Cranial nerve revisions.

Lautenschlager et al., 2012	Current Discussion
Not identified	Optic Nerve
Abducens Nerve	Oculomotor Nerve
Abducens Nerve	Trochlear Nerve
Trigeminal Nerve	Trigeminal Nerve
Taphonomic Distortion	Abducens Nerve
Pneumatic Diverticulum	Facial Nerve
Facial Nerve	Vestibulocochlear + Facial Nerve
Glossopharyngeal Nerve	Glossopharyngeal Nerve
Vagus Nerve	Vagus Nerve
Spinal Accessory Nerve	Spinal Accessory Nerve
Hypoglossal Nerve	Hypoglossal Nerve
Pituitary Chamber	Pneumatic Chamber

## Methods and materials

No permits were required for the described study, which complied with all relevant regulations. The *Nothronychus* braincase (AzMNH 2117) Arizona Museum of Natural History, Mesa, Arizona) was collected from the Turonian Moreno Hill Formation, Zuni Basin, New Mexico. It was described previously. The original specimens are stored at the Arizona Museum of Natural History, Mesa, Arizona.

## Description

The theropod skull has been frequently noted as extensively pneumatized [16], with cavities penetrating the facial bones and basicranium. The facial bones of *Nothronychus* are currently unknown, but it is likely that they were pneumatic, based on descriptions of other theropods [19]. The basicranium of *Nothronychus* is described as more highly pneumatic than is typical for theropods [10], but with respect to the median pharyngeal and subcondylar pneumatic systems, this description may be somewhat inaccurate. The development of the paratympanic sinus system, however, is extensively enlarged compared to other theropods, such as tyrannosaurids [20], ceratosaurs [21], and even that proposed for *Falcarius* [10]. In many cases, much of the basicranial pneumatic system, including the median pharyngeal and subcondylar systems, of *Nothronychus* and at least some other derived therizinosaurs such as *Erlikosaurus* [1] has been enclosed, probably by ossification of the associated respiratory epithelium around the air cavity or “ossified over” [9]. This development appears to be in process, but incomplete, in *Falcarius*.

**Fig 1:**
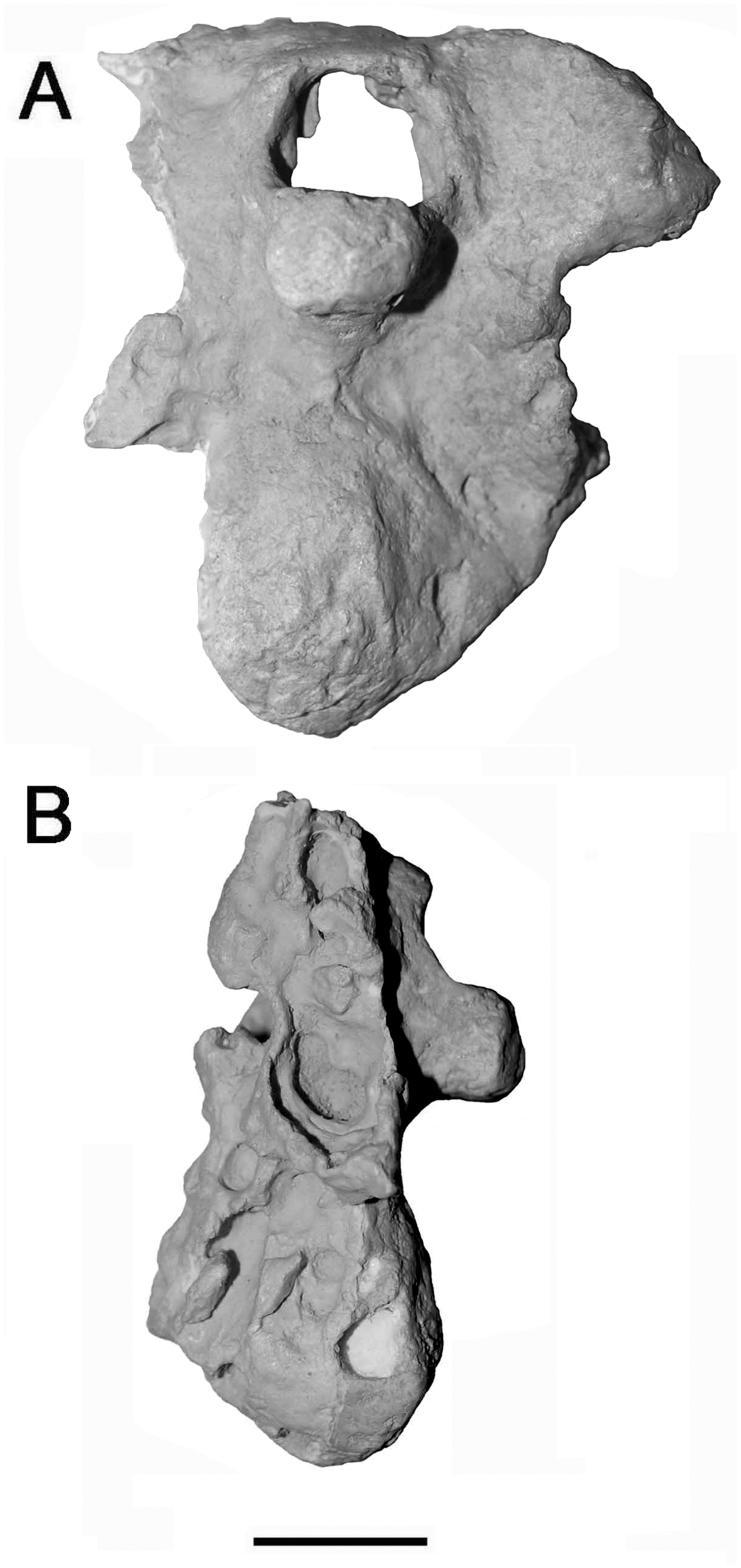
Basicranium of *Nothronychus mckinleyi* (AzMNH 2117) Cretaceous (Turonian) Moreno Hill Formation, Zuni Basin, West-Central New Mexico in A, posterior and B, left lateral views. Scale bar equals approximately 2 cm. Modified from Smith [10] with permission from Journal of Vertebrate Paleontology.

**Fig 2:**
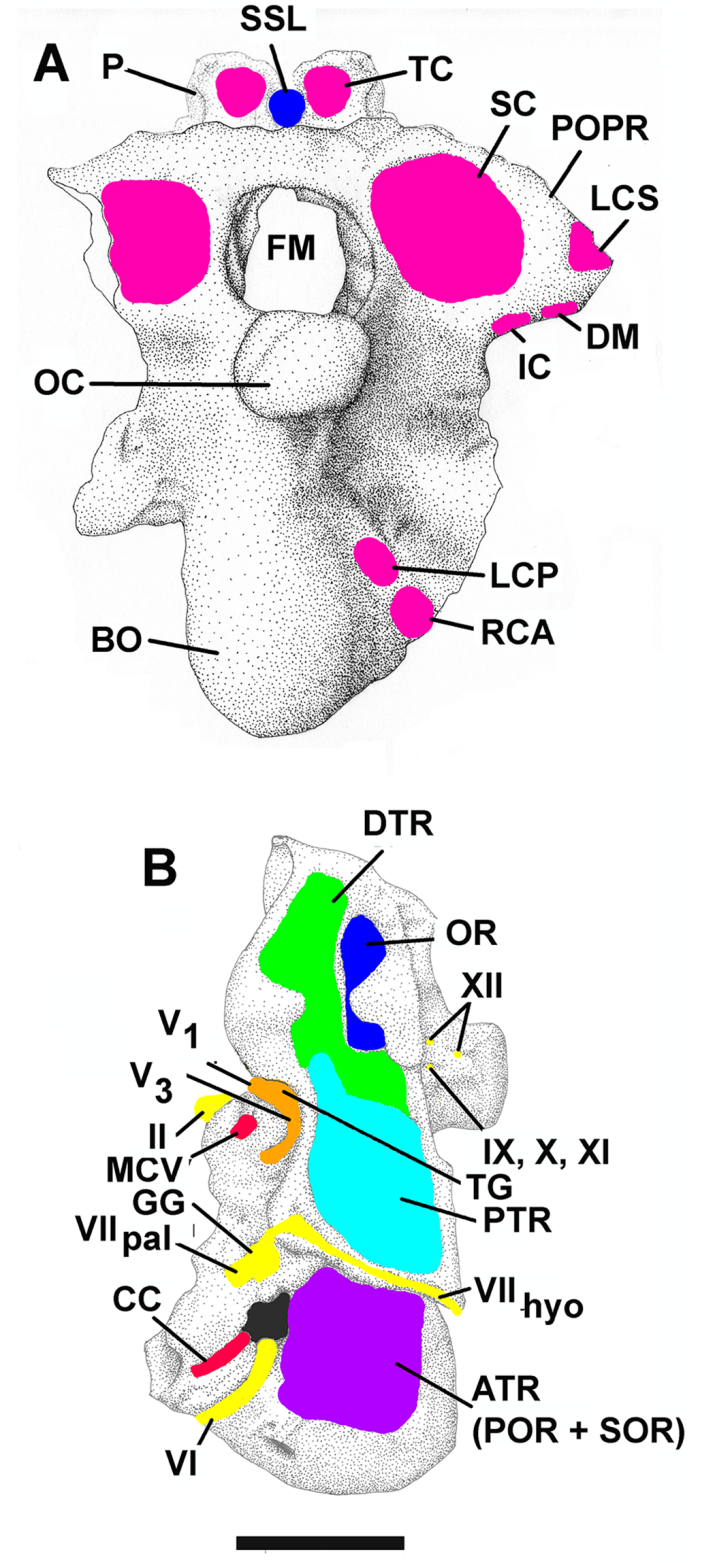
**Basicranium of *Nothronychus mckinleyi* (AzMNH 2117)** Cretaceous (Turonian) Moreno Hill Formation, Zuni Basin, West-Central New Mexico in A. posterior view. Pink represents bascranial muscle insertion points. Blue represents supraspinous ligament attachment point. and B. left lateral view. Blue/purple/green represents pneumatic spaces and the columellar recess, yellow nerves, and red venous structures. Scale bar equals approximately 2 cm. Modified from Smith [10] with permission from Journal of Vertebrate Paleontology.

The general osteology of the *Nothronychus mckinleyi* basicranium (AzMNH 2117) presented previously [10] is regarded as accurate (Figs 1 and 2). This description is predominantly based upon the better preserved left side where, however, some taphonomic compression is present compared to the right side. A posterior, unnamed, therizinosaur braincase, along with some other elements from multiple individuals, was recently uncovered from the Turonian Bissekty Formation of Uzbekistan [5] and was compared with *Nothronychus*. Osteologically, the braincases are quite similar dorsal to the occipital condyle, but results of a phylogenetic analysis [5] makes the Uzbekistan therizinosaur more primitive than the therizinosaurids *Erlikosaurus* and *Nothronychus*. As in *Nothronychus* [9], the supraoccipital is horizontally oriented in the Uzbekistan therizinosaur [5]. The Uzbekistan therizinosaur braincase is extensively pneumatized as expected, but it possesses open basisphenoidal and subcondylar recesses more similar to *Falcarius* [9] than *Nothronychus*. Another character shared with *Falcarius* is a ventral constriction in the neck of the occipital condyle and an overhanging occipital condyle. Like *Nothronychus* and in contrast to *Falcarius*, the Uzbekistan braincase lacks a pronounced condylotuberal crest. Some nervous structures have been revised (Table 1).

### Cranial nervous system

#### Optic nerve (II)

The location of the optic nerve very close to the optic chiasma (Figs 1 and 2) is given by Smith [10].

#### Oculomotor nerve (III)

**Fig 3:**
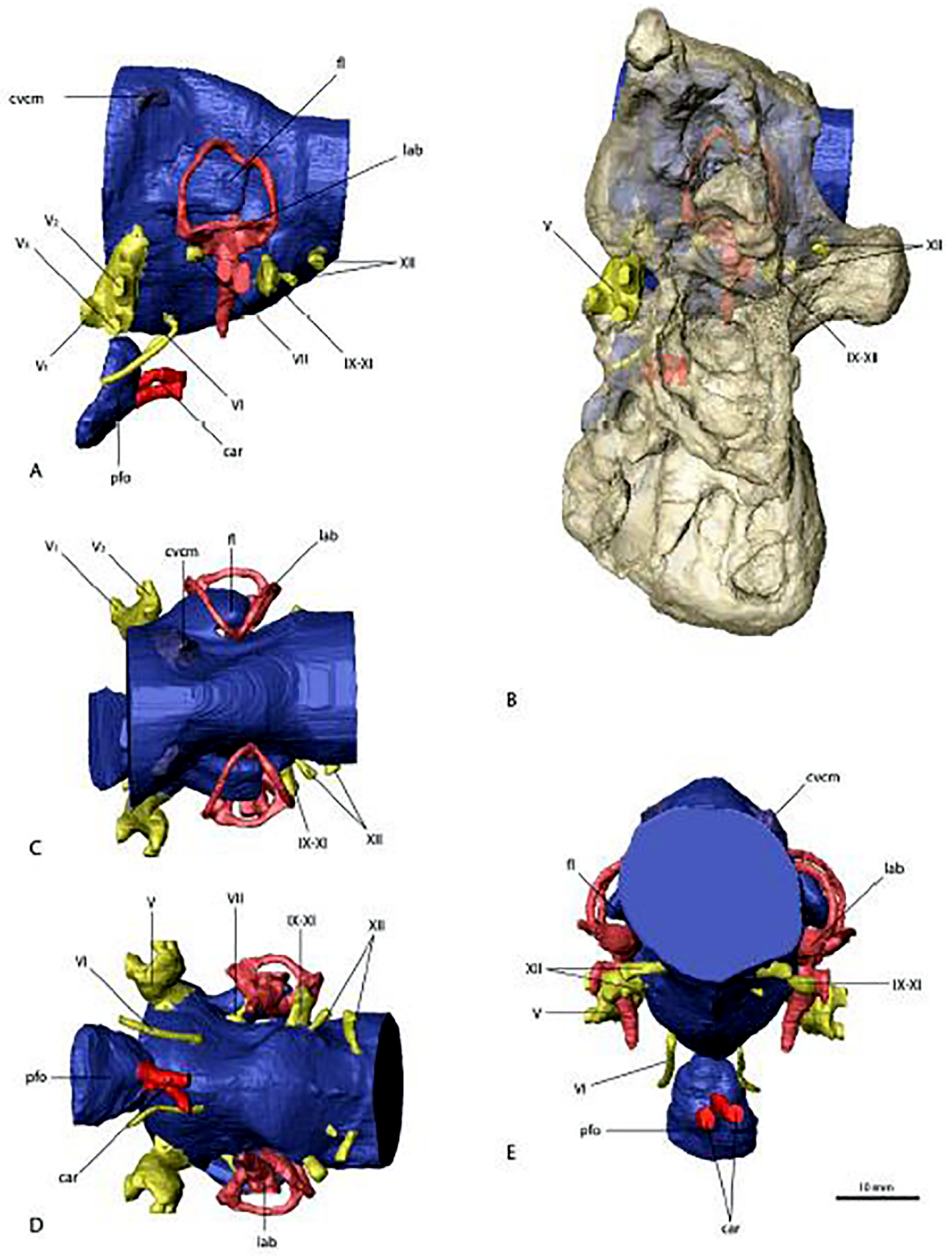
Reconstruction of Basicranial Soft Tissues of *Nothronychus mckinleyi* (AzMNH 2117) Cretaceous (Turonian) Moreno Hill Formation, Zuni Basin, West-Central in A, left; B, left with basicranium superimposed; C, dorsal; D, ventral; and E, anterior views. Blue represents endocranial cavity, Yellow represents cranial nerve tracts, dark red represents vascular and light red represents inner ear structures. Scale bar equals approximately 1 centimeter. After Lautenschlager et al. (2012) with permission from PlosOne.

The proposed identification of this nerve canal is suggested by Smith [10]. This reconstruction would reflect the primitive condition, where the oculomotor and trochlear nerves share a common canal, as seen in the braincase of *Allosaurus* [22]. See Lautenschlager et al., [7] for an alternative reconstruction (Fig 3), where this canal is reconstructed as transmitting the abducens nerve (VI) separate from nerve III.

#### Trochlear nerve (IV)

The possible identification of this nerve canal is indicated by Smith [10]. This reconstruction assumes that the observed (by CT-scan) dorsal internal chamber is pneumatic, housing an internal basiphenoidal sinus as described in the braincases of *Ceratosaurus* [21] and *Allosaurus* [22]. This cavity would probably then be homologous with an unlabeled sinus dorsal to the extensively pneumatized basisphenoidal bulla figured in the description of *Erlikosaurus* [2]. Support for this interpretation rests with the identification of the optic nerve (II) trace, and its close association with the infundibulum and pituitary gland in most tetrapods [23]. This topology, however, results in a very long infundibulum. See Lautenschlager et al. [7] for an alternative reconstruction (Fig 3), where this canal is interpreted as transmitting the abducens nerve (VI) and the associated internal chamber housed the pituitary gland and associated tissues. However, the chamber would be unusually posteriorly removed from the optic nerve in this reconstruction, whereas the typical condition in vertebrates is a much closer spatial association between the two [23].

#### Trigeminal nerve (V)

The trigeminal extends laterally through a large foramen that is partially preserved in the prootic (Fig 2). As is typical in vertebrates [23], three branches, the ophthalmic (V_1_), maxillary (V_2_), and mandibular (V_3_) branches, extend from a common trigeminal (Gasserian) ganglion. Lautenschlager et al. [7] are probably correct in modeling the close association of the ganglion with the large foramen bounded posteriorly by the prootic. The trunk leading to the ganglion from the brain is very short, particularly if it is nearly intracranial, as observed in extant birds and many coelurosaurs [20]. This hypothesized condition is regarded as derived [24]. The trunk was apparently even shorter, with three discrete small foramina associated with the individual rami of the trigeminal nerve in *Dromaeosaurus* [25], rather than single large one in *Nothronychus*.

The reconstruction of the ophthalmic nerve (V_1_) is not well-constrained in the available material of *Nothronychus*, but presumably diverged first, anteriorly from the trigeminal ganglion as in many derived theropods [20, 24]. It would thus be expected to have exited through a distinct foramen in the laterosphenoid separate from the combined maxillomandibular trunk (V_2-3_). Although this element is not preserved, it would have formed the anterior wall of the trigeminal foramen. This configuration appears to be present in the Uzbekistan therizinosaur [5]. The trunk composed of the combination of the maxillomandibular nerves was very short, as well, but was apparently longer in *Nothronychus* than in the Uzbekistan therizinosaur, where there are two trigeminal foramina [5]. An anterodorsal notch, rather than a ventral one, as in *Nothronychus* [10], probably transmitting the middle cerebral vein was described in the Uzbekistan therizinosaur [5]. A shallow anteriorly-directed groove extending ventral to the posterior margin of the trigeminal foramen is interpreted as indicating the trace of the mandibular nerve (V_3_). A short, deep second groove in the anterior face of the otosphenoidal crest extended laterally from the reconstructed trigeminal ganglion and reflected the posterior margin of the maxillary nerve (V_2_) before it, too, diverged from the braincase wall and proceeded anteriorly to innervate the maxilla [26].

#### Abducens nerve (VI)

This reconstruction follows that of Smith [10]. See Lautenschlager et al., [7] for an alternative pneumatic reconstruction (Fig 3).

#### Facial nerve (VII)

This reconstruction of the facial nerve places the major foramen ventral to the trigeminal foramen (Fig 2). The canal is not revealed in CT-scan, probably due to the aforementioned taphonomic compression. The placement of the facial nerve reflects the primitive condition for archosaurs, as seen in the braincase of *Allosaurus* [22]. See Lautenschlager et al. [7] for an alternative pneumatic reconstruction (Fig 3). In contrast to Smith [10], the geniculate ganglion is reconstructed as external to the prootic within a shallow lateral excavation as in *Majungasaurus* [24], rather than contained within the endocranial cavity, where space seems lacking. The trunk extends through a small foramen that is at least partially subdivided by a very thin vertically oriented lamina. This lamina may have, at least in part, divided the larger palatine ramus from the smaller hyomandibular ramus. There is no osteological correlate for the continued pathway of the palatine ramus, but the hyomandibular ramus is marked by a shallow groove in the margin of the otosphenoidal crest following Smith [10], similar to *Majungasaurus* [24] and modern varanid lizards [27].

#### Vestibulocochlear nerve (VIII)

This reconstruction follows Smith [10] in that one small foramen directed towards the cochlea is reconstructed to have transmitted a single, vestibulocochlear nerve along with some fibers of the facial, as is typical for vertebrates [26]. This morphology is in contrast to that described for other theropods including *Tyrannosaurus* [28], *Troodon* [29], *Velociraptor* [30], and *Incisivosaurus* [31] where separate foramina transmitted discrete cochlear and vestibular nerves and a third foramen transmitting facial nerve fibers. The presence of the other two foramina may have been taphonomically concealed in the *Nothronychus* braincase, but the possession of a single united foramen is also apparent in *Falcarius* [9]. Fusion of the vestibulocochlear foramina with the facial into a single foramen would be highly unusual for theropods and may be apomorphic for therizinosaurs. In any case, it is probable that some fibers of the facial were either transmitted through the single observed foramen or in a discrete, taphonomically destroyed foramen to the inner ear [26], but most would have followed a separate pathway in a discrete foramen to the external wall of the prootic as in other archosaurs [32, 33]. See Lautenschlager et al. [7] for an alternative reconstruction (Fig 3) that does not recognize a discrete vestibulocochlear canal. In this latter interpretation, the single observed foramen would have transmitted the facial nerve.

#### Glossopharyngeal (IX), Vagus (X), Spinal Accessory (XI), and Hypoglossal (XII) nerves

The remaining cranial nerves (Fig 2) were similarly reconstructed for *Nothronychus* by Lautenschlager et al. [7] and Smith [10].

#### Pneumatic sinuses

The basicranial pneumatic system of therizinosaurs (Fig 2) has often been characterized as being very extensive [9, 10], but the basisphenoidal and subcondylar systems may only be apparently exaggerated in that they are enclosed by bone and are actually little or no more extensive than in other theropods, such as tyrannosaurs [28, 33]. The only unusually enlarged pneumatic cavity relative to other coelurosaurs, then, would be the paratympanic system. Notably, the tympanic recess is not “ossified over”, unlike the other two systems. Following Witmer and Ridgely [20], cavities representing the paratympanic system are externally visible without CT-scan. There is a cavity lateral to the basisphenoidal bulla that is referred to the anterior tympanic recess (combined prootic and subotic recesses following the terminology of Witmer [19]. This recess is apparently shifted somewhat ventrally, below the otosphenoidal crest. Communication between the contralateral cavities (referred to as the retrohypophyseal sinus by Witmer [19] is uncertain. The deep ventral cavity is identified as the posterior tympanic recess. This chamber extends into the paroccipital process [7], as expected for maniraptoran theropods and similar to the Uzbekistan therizinosaur [5]. It is partially continuous with the possible dorsal tympanic recess that is anterior, extending dorsal, to the middle ear. As in tyrannosaurs [20], the posterior tympanic recess is the largest basicranial pneumatic recess in *Nothronychus*. These tympanic recesses are closely related to the middle ear [20]. Witmer and Ridgely propose that such an expansion in the tympanic system has an extensive influence on low frequency sound sensitivity in many theropods, including *Nothronychus*. The tympanic system is notably larger in *Nothronychus* than *Falcarius* [9], so perhaps low frequency sound sensitivity was enhanced, as well. As noted for tyrannosaurs [20], there is some side-to-side variation in the basicranial pneumaticity of both *Falcarius* and *Nothronychus*. The cavities making up the median pharyngeal and subcondylar systems are probably present in *Nothronychus*, as in *Falcarius* [9] and some other coelurosaurs [24], but would require CT data to observe, as they are enclosed in bone [10]. Some cavities are directly visible in breaks in the basicranium of *Erlikosaurus* [2] that probably correspond to these systems. The corresponding surrounding ossification would seem to indirectly support the origin of the subcondylar recess from a tympanic air sac [34] or an extension of the basisphenoidal recess [35], rather than a pulmonary diverticulum extending from the cervical vertebrae as proposed by Witmer [19]. However, the embryological origin of the basicranium from anterior cervical somites presented here would support Witmer’s suggestion. The basisphenoidal and subsellar recesses are included within the median pharyngeal system, following Witmer and Ridgely [20]. These, along with the dividing transverse lamina are contained within the basisphenoidal bulla and not visible externally without CT-scan. The associated cavities are probably derived from a separate diverticulum from the pharynx as proposed by Witmer [19], and Sampson and Witmer [24] but then closed off in *Nothronychus* and *Erlikosaurus* [2].

### Craniocervical musculature and the supraspinous ligament

The craniocervical musculature of *Nothronychus* is modeled on that proposed for the tyrannosaurids and other large theropods [36]. The supraoccipital of *Nothronychus* appears undistorted, oriented horizontally, and lacking a nuchal crest (Figs 1 and 2) [10], very similar to the Uzbekistan therizinosaur [5]. Rather than inserting on the supraoccipital [11], the supraspinous ligament and m. transversospinalis capitis are reconstructed as partially passing dorsal to that element to insert on the currently unavailable parietal hypothesized to be expressed as a small crest in this region. Therefore, the supratemporal fenestra and associated origins for the mandibular adductor musculature are shifted roughly two centimeters anteriorly from the occiput, an arrangement not observed in other theropods, including *Allosaurus* [22], or the therizinosaurs *Falcarius* [9] and *Erlikosaurus* [7]. This architecture is unusual for a theropod in that the insertion is far anterior, but is constrained by the horizontal supraoccipital possessed by *Nothronychus*. This insertion point was apparently similar in the braincase of the Uzbekistan therizinosaur. Only further discoveries will resolve the question regarding the configuration of the parietal.

## Discussion

### Soft tissue reconstruction update

The relationship of the parietal with the supraoccipital has undergone some re-evaluation. As a result, the proposed insertion points for the dorsiflexive muscle m. transversospinalis capitis and the supraspinous ligament are somewhat altered from Smith [11]. As a result of the enlarged middle ear, the hypothesized parietal crest and associated supratemporal fenestra are shifted unusually far anteriorly. Therefore, m. transversospinalis capitis partially extends dorsally over the horizontal supraoccipital to reach the parietal crest. This change may have an effect on previous functional interpretations of the dorsiflexive capability of m. transversospinalis capitis [11] as the insertion is moved away from the occipital condyle, thereby increasing power and reducing speed of dorsiflexion. The ventroflexive and lateroflexive muscle groups and functions are unaffected (Fig 2). The cranial nervous system interpretation (Fig 2) for *Nothronychus* is altered from Lautenschlager et al. [7] and Smith [10]. Lautenschlager et al.’s [7] interpretation is probably correct in the placement of the trigeminal nerve (V). *Nothronychus* shared the derived condition with *Tyrannosaurus* and extant birds [20] in having a very short trunk and internal trigeminal ganglion. The ophthalmic branch (V_1_) would have projected anteriorly from this point as described for *Tyrannosaurus* [20]. A separate foramen for the ophthalmic nerve would be predicted. Therefore, an extracranial divergence between the ophthalmic and maxillomandibular branches as seen in *Majungasaurus* [24] would be unexpected using phylogenetic inference and available osteology. The union of the mandibular and maxillary branches (V_2-3_) is very short, before the mandibular branch diverges through a shallow external groove extending anteriorly dorsal to the geniculate ganglion. From the trigeminal ganglion, the maxillary branch extended laterally, marked by a deep groove in the otosphenoidal crest, before turning anteriorly towards its target. This pattern is present in some theropods (e.g. *Tyrannosaurus* and birds, according to Witmer et al. [33] but not all (e.g. *Majungasaurus*, [24]). Sampson and Witmer [24] regard the intracranial trigeminal ganglion as derived.

The geniculate ganglion associated with the facial nerve (VII) appears to be immediately adjacent to the prootic and ventral to the trigeminal. This would be a typical architecture for archosaurs. Therefore, the interpretations by Lautenschlager et al. [7] and Smith [10] for this region would both probably be incorrect. Using the architecture of other theropods (e.g. *Ceratosaurus* [21]), it would appear that taphonomic distortion may have collapsed the internal nerve tracts on the left side. No further alteration from Smith [10] can be supported as the nerves must have exited through the prootic. Exits through the laterosphenoid would be highly unusual for archosaurs. There is no evidence for such architecture in the available material. The foramen leading to the cochlea probably mainly transmitted vestibulocochlear (VIII) fibers with only a minor facial (VII) nervous component, based on descriptions of other theropods such as *Allosaurus* [22] and modern tetrapods [26].

### Pneumatic sinuses in *Nothronychus*

Tympanic Sinuses, the Cochlea, and Low-Frequency Hearing. The columellar recess in *Nothronychus* is very similar to that described for the alvarezsaurs *Shuvuuia* and *Mononykus* [37]. The columella (stapes in mammals) followed a groove in the paroccipital process, but did not perforate it, similar to what is seen in the two alvarezsaurs.

While frequency sensitivity is closely linked to the length of the basilar papilla (organ of Corti in mammals) (best fit line [38]

y=5.7705e-0.25x,r=0.96)38, (1)

the presence of these tympanic sinuses would suggest a much lower frequency range than predicted by extrapolation of their regressions. While basilar papilla length correlates well with mass in birds

y=3.18ln(x)+3.5228,r=0.87 (2)

as reported by Gleich et al., [38], the best frequency of hearing as a function of mass is less predictable. The presented calculated correlation coefficient (r) of 0.74 for the best fit line

y=2.2582x-0.1016 (3)

between best frequency sensitivity and body mass in birds [38], while notable, still shows a considerable range of error, reducing the applicability to larger animals. In both regressions, the low frequency sensitivity increases with increasing body mass and organ of Corti length.

Increased vocal complexity is related to sociality in mammals such as squirrels [39] and birds including penguins [40]. Walsh et al. [41] observed a correlated increase in the length of the cochlea, vocal complexity, and sociality in birds. Presumably, this development would be associated with an increase in communication and auditory complexity of the environment as noted by Lautenschlager et al., [7]. However, the increase in cochlear length was present in many coelurosaurian theropods including tyrannosaurs and ornithomimids [20], implying that sociality may not have been uncommon in derived theropods. Therefore, a long cochlea in *Nothronychus* would have been plesiomorphic for that genus.

The pneumatic chambers extending to the external braincase include the anterior (rostral) tympanic recess (merged prootic and subotic recesses), and the confluent dorsal and posterior (caudal) tympanic recesses (Fig 2) [19]. The presence of enlarged pneumatic chambers associated with the middle ear is closely associated with enhanced low frequency sensitivity in other reptiles and birds [20, 40, 41, 42], possibly including an infrasound capability. The increased volume has been shown to decrease impedance on the columella and permit sound amplification [20, 43] and references therein). This capability might have been present in *Nothronychus*. Some birds, such as pigeons, have even been shown to be sensitive to very low frequencies (infrasound frequencies less than 100 Hz) resulting from specialized cells at the apex of the organ of Corti [44, 45]. Therefore, the calculated best frequency sound sensitivity of 1100 to 1450 Hertz and upper limits of 3000 to 3700 Hertz in *Nothronychus* [7] using the regressions of Gleich et al. [38] must be considered high estimates. Frequency calculations based on cochlear length agreed well with observed results in extant penguins [40], but they noted the reduction of the paratympanic sinuses in many diving birds. This development logically might reduce their contribution to low frequency sound sensitivity, but has not been tested.

The expanded pneumatic chambers were originally observed in *Nothronychus* by Smith [10], but no function was proposed at the time. A possible infrasound capability is often associated with communication and navigation [44, 45]. The presence of these sinuses would have resulted in optimal sound frequency reception quite a bit lower than that modeled by Lautenschlager et al. [7] based on the cochlear length alone, but the contribution of the tympanic sinuses complicates Hertz inferences for any extinct maniraptoran theropods. Proposed functional applications include long distance navigation and intraspecific communication [45]. Long distance navigation seems unlikely for these animals, so enhanced intraspecific communication ability is preferred here as seen in many other archosaurs [44].

### Subcondylar and middle pharyngeal sinuses

Ossification of the epithelium associated with the subcondylar and middle pharyngeal sinuses, but not the tympanic system, in derived therizinosaurs might be explained embryologically. This development does not appear analogous to the development of the auditory bullae from the fused tympanic, anterior entotympanic, and posterior entotympanic in mammalian Carnivora and is related to increased sound sensitivity [46]. There is no apparent association of the middle pharyngeal sinus with the middle ear in therizinosaurs. In therizinosaurs, it may have resulted from the altered and increased stress fields associated with herbivory [19]. However, this pattern does not account for pneumatic patterns observed resulting from herbivory as proposed in some other theropods, such as oviraptorosaurs and ornithomimosaurs. Additionally, the braincases of widely accepted herbivorous dinosaurs, such as the ceratopsians (e.g. *Zuniceratops* [47] and hadrosaurs (e.g. *Hypacrosaurus* [48]) lack extensive basicranial or vertebral pneumatization. An alternative hypothesis is that this development was independent of any stress fields induced by herbivory in small-headed taxa. In this case, as in extant anserines and ratites that have extensively pneumatized basicrania, the stresses produced would have been insignificant as most food processing would presumably have occurred in a gizzard and not associated with oral mastication. Therefore, like in sauropods, geese, and ostriches, therizinosaurian mouth function is mainly associated with food gathering and not processing [49].

**Fig 4:**
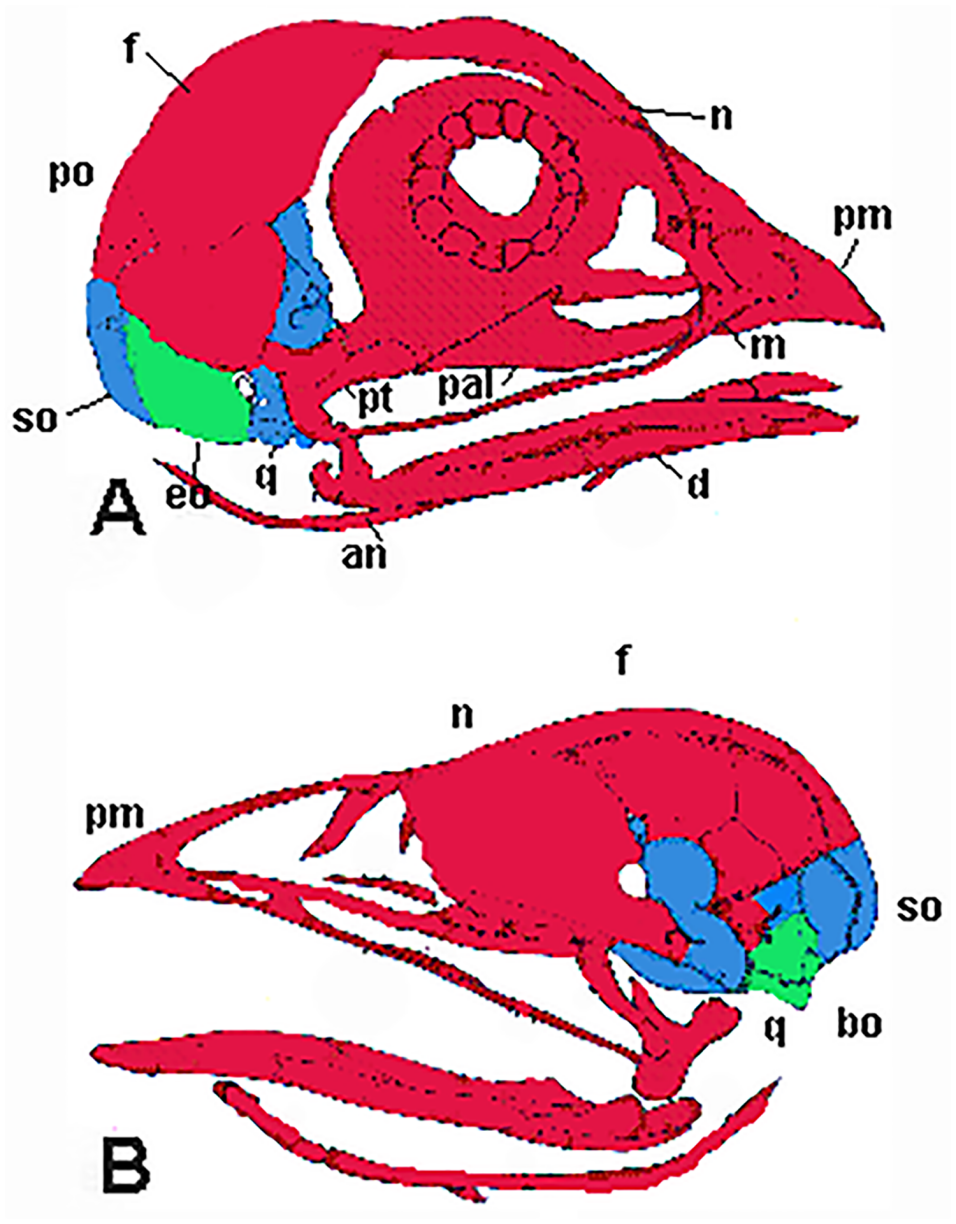
Schematic extant bird skull in A, right lateral and B, right internal view. Red represents skull originating from neural crest, blue, cephalic mesoderm origin, and green cephalic mesoderm origin. Modified from Couly, Coltey, and Le Douarin (1993) with permission from Development.

**Fig 5:**
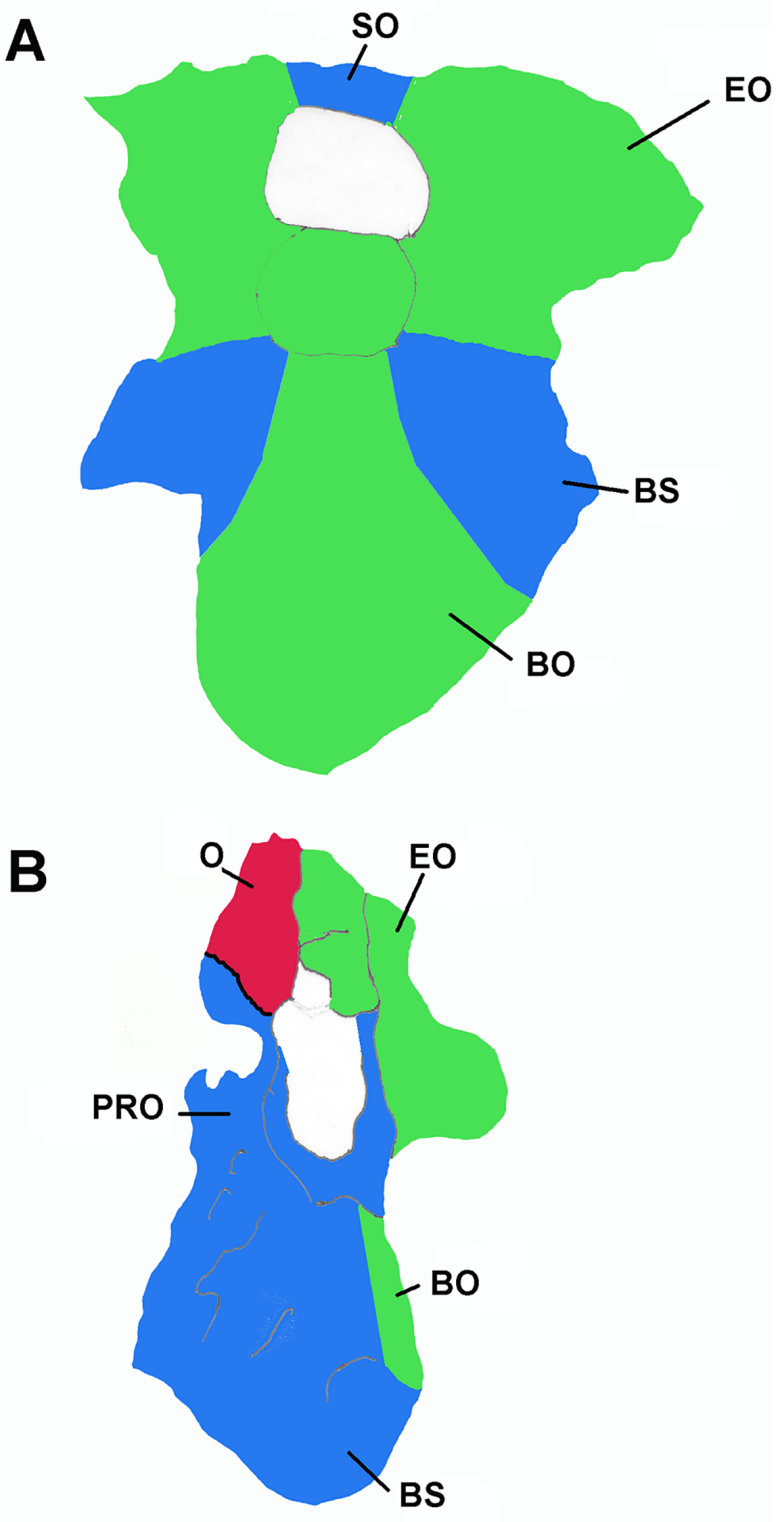
Schematic *Nothronychus mckinleyi* braincase in A, lateral and B, posterior views. Red represents skull originating from neural crest, blue, cephalic mesoderm origin, and green cephalic mesoderm origin. Modified from Smith [10] with permission from Journal of Vertebrate Paleontology. Scale bar equals approximately 2 cm.

**Fig 6:**
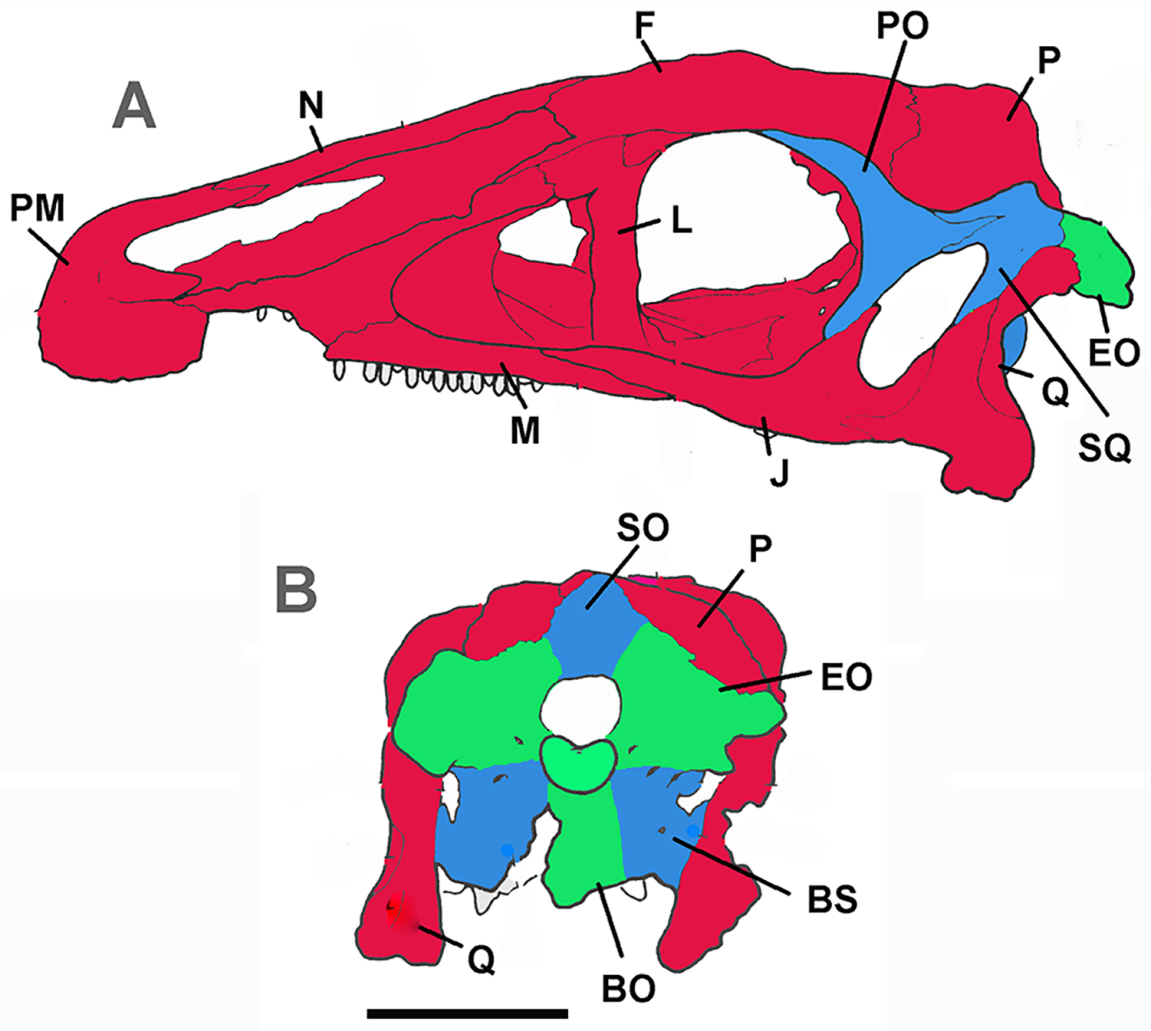
Schematic *Erlikosaurus andrewsi* skull in A, lateral and B, posterior views. Red represents skull originating from neural crest, blue, cephalic mesoderm origin, and green cephalic mesoderm origin. Modified from Clark, Perle, and Norell, 1994 with permission from American Museum of Natural History. Scale bar equals approximately 5 cm.

Development of the basicranium, including pneumatization, should mirror that of the cervical spine, since the basicranium is embryologically and evolutionarily derived from the cervical sclerotomal and myotomal elements [18, 50, 51]. Experimental results based on quail and chick development describe a tripartite origin for the vertebrate skull (Fig 4) [18, 50]. Piekarski et al. [52] provide evidence that skull developmental patterns are primitive for tetrapods, except at least some anurans. Therefore, they are probably similar for any theropod, including birds, and can be mapped onto the skulls of *Nothronychus* (Figs 4 and 5) and *Erlikosaurus* (Fig 6). Most of the skull is derived from neural crest cells but evolutionary trends in vertebrates incorporate increasing numbers of anterior cervical somites into the basicranium [50]. The first five somites fuse, forming the basisphenoid. Somite development is regulated by Hox genes, with individual vertebrae incorporating anterior and posterior regions of successive somites [51]. Therefore, the occiput of any vertebrate is developmentally reminiscent of an anterior cervical vertebra, with the exoccipital and supraoccipital representing the neural arch and the basioccipital, the vertebral centrum [50]. This embryological information would seem to support the identification of the posterior internal chamber within the basicranium as pneumatic, rather than housing the pituitary gland.

Therizinosaurs are characterized by highly pneumatic cervical vertebrae [53]. The observed morphology in *Nothronychus*, then, is the result of the genetic control of the osteological interplay with the diverticular pneumatic epithelium of the basicranium. Differential,—increased, expression of a series of Hox genes would result in pneumatic, enclosed chambers within the basicranium similar to the highly pneumatic anterior vertebral centra. Any selective advantage of this development is unclear. The possibility remains that this ossification is selectively neutral. Activation or increased expression of the regulating Hox gene may have become fixed due to possible proximity to a selected gene.

In large ratites, there is a tendency towards extreme postcranial pneumatization and ossification of the epithelium would be associated with general cameral pneumatization about vertebral air sacs, presumably associated with body mass reduction [53]. In *Erlikosaurus*, the basisphenoidal bulla is subdivided by septa [2] into a series of larger and smaller chambers. If the specimen was a cervical vertebra, these resulting chambers would be referred to as larger camera and smaller camella [50, 54, 55]. Since the basicranium is embryologically homologous with cervical vertebrae [48, 49], the internal development of this region would probably be similar, with camera associated with small camellae. The process is in contrast to somphospondylous pneumatization, where a pneumatic epithelium expands inside the bone, resulting in much smaller air spaces as in *Euhelopus* [55] or the camerae observed in the vertebrae of brachiosaurid sauropods [54, 55, 56, 57] and most large theropods [54].

### Vestibular apparatus in therizinosaurs

Both *Nothronychus* and *Falcarius* retain the elongate semicircular canals [7, 9] of their predatory relatives (e.g. *Tyrannosaurus*). The relationship of the semicircular canals and flocculus to gaze stabilization and general eye movement, including tracking (vestibulo-ocular and vestibulocollic reflexes) has often been noted [33, 58]. This semicircular canal configuration is apparently primitive for therizinosaurs as briefly noted by Lautenschlager et al. [7], but this elongation and retention has been correlated with an active, agile life style of predators, rather than the slow moving herbivorous niche that has occasionally been proposed [59]. This observation, however, should not be taken as an argument that *Nothronychus* was an active predator, but merely that it retained the ancestral ear configuration. As a possible amphibious animal, selection may not have modified the ear configuration in this area.

As presented for *Erlikosaurus* [7], *Nothronychus* probably had a nearly horizontal head posture, suggested to be associated with overlapping visual fields and binocular vision, based on the orientation of the horizontal semicircular canal relative to the horizontal orientation of the occipital condyle. However, a considerable amount of variability in this trait has been observed among carnivorous taxa [33].

## Conclusions

The soft-tissue inferences for the basicrania of *Nothronychus* and, to some extent, *Falcarius* have been re-evaluated as a result of new information and continued study. In some cases, such as the cranial nerve reconstruction, there is apparently little functional effect, but there are implications for theropod evolution. The results presented here bring *Nothronychus* more in line with derived maniraptoran theropods, giving the therizinosaur basicranium typical trigeminal and facial nerve morphologies [24], although the facial foramen is displaced ventrally. The dorsiflexive craniocervical musculature insertions were moved anteriorly and there may be some effect on the resulting lever arms, but this has yet to be evaluated.

The development of the basicranium of *Nothronychus* may not be associated with stress field resulting from herbivory, but related to vertebral development as the base of the skull is derived from the anteriormost cervical somites (Fig 3). Ossification of the basioccipital and basisphenoid and the associated pneumaticity can all be derived from anterior cervical vertebral structures. Therefore, the development of the therizinosaur basicranium reflects the tripartite origin of the vertebrate skull (Figs 3–5).

Optimal sound frequencies for *Nothronychus* and *Falcarius* [7] may be overestimated due to the contribution of the tympanic sinuses that would tend to decrease the stiffness of the columella [20, 42]. Finally, cochlear length in both therizinosaurs has been correlated with an increase in sociality [6, 41]. This concept is supported, but not confirmed by the preservation of *Falcarius* in a bone bed [4].

## Abbreviations

**ATR**: Anterior Tympanic Recess
**AzMNH**: Arizona Museum of Natural History, Mesa, Arizona
**BO**: Basioccipital
**BPP**: Basipterygoid Process
**BS**: Basisphenoid
**BSB**: Basisphenoidal Bulla
**BT**: Basal Tubera
**CC**: Carotid Canal
**CR**: Columellar Recess
**Cranial Nerves**: II, V_1-3_, VI, VII, IX, X, XI, XII
**DM**: m. Depressor Mandibulae
**DTR**: Dorsal Tympanic Recess
**EO**: Exoccipital
**F**: Frontal
**FM**: Foramen Magnum
**GG**: Geniculate Ganglion (Cranial Nerve VII)
**hyo**: hyomandibular branch (Cranial Nerve VII)
**IC**: m. iliocostalis capitis
**IOS**: Interorbital Septum
**J**: Jugal
**L**: Lacrimal
**LCP**: m. Longissimus Capitis Profundus
**LCS**: m. Longissimus Capitis Superficialis
**M**: Maxilla
**MCV**: Middle Cerebral Vein
**N**: Nasal
**O**: Opisthotic
**OC**: Occipital Condyle
**OP**: Opisthotic
**OR**: Otic Recess
**P**: Parietal
**PAL**: palatine
**pal**: palatine branch (Cranial Nerve VII)
**PM**: Premaxilla
**PN**: Pneumatic Space
**POPR**: Paroccipital Process
**POR**: Prootic Recess
**PRO**: Prootic
PT: Pterygoid
**PTR**: Posterior Tympanic Recess
**Q**: Quadrate
**RCA**: m. Rectus Capitis Anterior
**SC**: m. Splenius Capitis
**SO**: Supraoccipital
**SOR**: Subotic Recess
**SQ**: Squamosal
**SSL**: Supraspinous Ligament
**TC**: m. Transversospinalis Capitis
**TG**: Trigeminal (Gasserian) Ganglion
